# Self-assembled Janus base nanotubes: chemistry and applications

**DOI:** 10.3389/fchem.2023.1346014

**Published:** 2024-01-18

**Authors:** Wuxia Zhang, Yupeng Chen

**Affiliations:** Department of Biomedical Engineering, University of Connecticut, Storrs, CT, United States

**Keywords:** hydrogen bond, stacking, Janus base nanotubes, nanotechnology, scaffold, RNA delivery, sensor

## Abstract

Janus base nanotubes are novel, self-assembled nanomaterials. Their original designs were inspired by DNA base pairs, and today a variety of chemistries has developed, distinguishing them as a new family of materials separate from DNA origami, carbon nanotubes, polymers, and lipids. This review article covers the principal examples of self-assembled Janus base nanotubes, which are driven by hydrogen-bond and π-π stacking interactions in aqueous environments. Specifically, self-complementary hydrogen bonds organize molecules into ordered arrays, forming macrocycles, while π-π interactions stack these structures to create tubular forms. This review elucidates the molecular interactions that govern the assembly of nanotubes and advances our understanding of nanoscale self-assembly in water.

## 1 Introduction

The chemistry of DNA base pairs offers a captivating journey into the molecular intricacies that underlie the genetic code of life. Comprising adenine (A), thymine (T), cytosine (C), and guanine (G), these base pairs constitute the essential rungs of the DNA double helix. Governed by hydrogen bonding, the interactions between these bases create a remarkably stable and specific pairing mechanism: A pairs with T, and C pairs with G. This precise complementary bonding ensures the faithful replication of genetic information during cellular processes. The elegant interplay of chemical forces within DNA base pairs not only upholds the integrity of our genetic material but also serves as a wellspring of inspiration for innovative materials like Janus base nanotubes, where the principles of DNA chemistry find novel expression in the realm of nanotechnology.

Hydrogen bond is a fundamental force of attraction between hydrogen atoms and electronegative atoms such as oxygen, nitrogen, or fluorine (Jeffrey, 1997). In the context of self-assembly in water, solvent molecules can compete effectively for hydrogen bonds (Nagy, 2014), so it becomes crucial to engineer the structures of monomers so that hydrogen bond pairs can be formed among monomers rather than with water molecules. In natural DNA, the structure of hydrophobic heterocycles possessing hydrogen bond donors/acceptors effectively shields the hydrogen bonding from water (Feng et al., 2019). The specific recognition of complementary hydrogen bond pairs allows for the correct assembly of 2D structures (Sijbesma et al., 1997).

In addition to hydrogen bonding, another critical element in the self-assembly of tubular structures is π-π stacking (Martinez and Iverson, 2012). This non-covalent interaction occurs between the aromatic rings of molecules, characterized by the attractive forces between the π clouds. π-π stacking drives the assembly of 2D structures into well-defined, elongated 3D structures (Meng et al., 2020), ultimately giving rise to nanotubular architectures.

While numerous examples (Kimizuka et al., 1995; Marsh et al., 1996; Kolotuchin and Zimmerman, 1998; Klok et al., 1999; Whitesides et al., 2002; Mascal et al., 2003) of nanotubular structures have been successfully formed in organic solvents, this review focuses on aqueous environments, traditionally considered challenging for organized nanomaterial formation. This interesting combination of hydrogen bonding, π-π stacking, and self-assembly in water provides a potential way for designing novel water-soluble nanomaterials.

Nanomaterials have given rise to a new generation of highly efficient and compact sensor devices (Willner and Vikesland, 2018). Their ample surface area and customized electronic properties enable rapid and selective interactions with target analytes and chirality recognition (Salikolimi et al., 2020), making them indispensable in environmental monitoring, healthcare, and various industrial domains. Their adaptability and responsiveness have expanded the horizons of sensor technology, promising innovations that enhance our capacity to monitor, manage, and comprehend our surroundings.

In the realm of biotechnology, nanomaterials have emerged as versatile and potent tools, leveraging their unique attributes at the nanoscale to drive a broad spectrum of applications. These include targeted drug delivery systems (Chen et al., 2011; Yau et al., 2021a; Vargason et al., 2021), cutting-edge imaging agents for disease diagnostics (Chapman et al., 2013; Han et al., 2019), inventive tissue engineering scaffolds (Zhou et al., 2020; Zhou et al., 2021a; Yau et al., 2021b; Zhang et al., 2021), and high-performance biosensors for swift disease detection (Pirzada and Altintas, 2019; Yau et al., 2023). Their ability to engage with biological molecules and structures at a fundamental level holds immense potential for optimizing the precision, efficiency, and efficacy of various biotechnological endeavors, ultimately pushing the frontiers of medical science and biomedicine.

In this review, the design and characterization of different Janus base nanotubes will be discussed, along with significant properties and applications.

## 2 Chemical design and characterization

### 2.1 Dimeric building block

**FIGURE 1:**
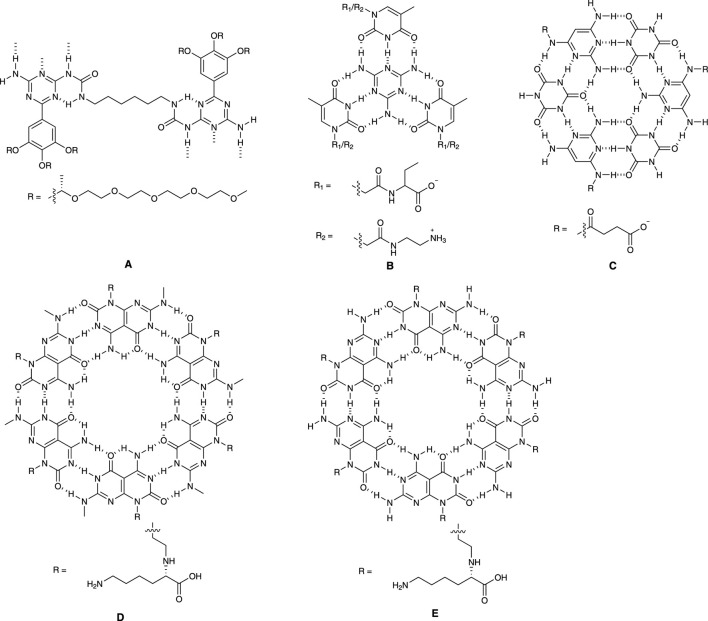
Cross section of different nanotubes.

Meijer and colleagues (Hirschberg et al., 2000) designed functionalized monomer unit (Figure 1A) which can self-assemble into non-covalently linked columnar polymeric structures. By tuning the side chains, the helicity of these structures can be adjusted.

The authors found that all compounds exist as individual molecules in dimethyl sulfoxide due to its interference with the hydrogen bonding. In contrast, when placed in chloroform, these compounds assemble into viscous solutions. The researchers used techniques like circular dichroism (CD) spectroscopy and small-angle neutron scattering (SANS) to study the properties of these structures. CD spectroscopy revealed that in dodecane, the molecules formed helical arrangements, and these helical structures can be denatured with high temperature or solvents such as chloroform and hexane. SANS experiments confirmed the presence of columnar structures in deuterated dodecane.

Furthermore, the researchers explored the formation of similar columnar structures in water. They designed molecules with chiral ethylene oxide side chains to achieve solubility in water and found that these molecules could form helical columns in water as well and their helicity is influenced by the peripheral chiral side moieties.

Overall, the researchers determined that the supramolecular polymer backbone is necessary to form stable, well-defined helices. The stacking of the hydrogen-bonded pairs leads to hydrophobic microdomains, allowing cooperative hydrogen bonding and transmission of the peripheral chirality into the helix.

### 2.2 Tetrameric building block

Xiao and colleagues (Xiao et al., 2018) developed a system (Figure 1B) containing two derivatives derived from thymine (T), specifically N-[2-(3,4-Dihydro-5-methyl-2,4-dioxo-1(2H)-pyrimidinyl)acetyl]-L-phenylalanine (T-phe) and N-(2-Aminoethyl)-3,4-dihydro-5-methyl-2,4-dioxo-1(2H)-pyrimidineacetamide (T-NH_2_). Then, they explored the optimal conditions for the self-assembly of T-phe and T-NH_2_ driven by melamine (M).

The researchers tested various combinations of these compounds and identified the optimal ratio was M:T-Phe:T-NH_2_ = 1:1.5:1.5. This ratio resulted in the most efficient recognition and base stacking and strongest attraction among T-Phe, T-NH_2_ and M. Additionally, the study examined how changes in pH affected the self-assembly process. Notably, the largest assemblies formed at a neutral pH of 7.2 when both T-Phe and T-NH_2_ were in their charge state. The researchers also investigated the concentration of the compounds and found that a critical concentration of 0.03 mM was necessary for self-assembly to occur. Microscopic images revealed that the assemblies had a fibrous structure with a diameter between 2.6 and 3.2 nm.

The study proposed that the self-assembly process followed a cooperative nucleation-growth pathway, where tetrameric structures initially formed through hydrogen bonds and were then extended through π-stacking and electrostatic interactions. Electrospray ionization mass spectrometry (ESI-MS) suggested the process was more likely to involve synergistic self-assembly rather than alternate self-assembly.

Lastly, the research demonstrated that these assemblies could form hydrogels at high concentrations, which could have practical applications in biomaterials and supramolecular polymer design.

### 2.3 Hexameric building block

Hud and colleagues (Cafferty et al., 2013) designed a water-soluble self-assembly system (Figure 1C) employing two low-molecular-weight monomers: cyanuric acid (CA), and a modified version of triaminopyrimidine (TAP), specifically, succinate-conjugated 2,4,6-triaminopyrimidine (TAPAS). The modification of TAPAS was intended to prevent the formation of undesired structures and improve solubility of desired assemblies.

When the concentration of the CA and TAPAS mixture in aqueous solution reached 3.5 mM at 1:1 ratio, the rise of UV absorption at 320 nm indicated ring stacking and the mixture started to form gel above 5 mM. These assemblies were observed using atomic force microscopy (AFM) and transmission electron microscopy (TEM). Single fibers had a diameter of about 1.5–2.2 nm, and there were also aggregates observed, contributing to the formation of larger networks, which is consistent with gel formation. Nuclear Magnetic Resonance (NMR) spectroscopy was used to study how CA and TAPAS associated with each other at different concentrations. The results showed that the formation of TAPAS-CA assemblies was a highly cooperative transition in which only individual monomers and large assemblies coexist without any intermediate structures. Dynamic light scattering (DLS) further supported this observation, showing a dramatic increase in scattering intensity when the concentration of each monomer was above 3.5 mM.

The study also compared these structures to those found in RNA and DNA. The findings suggest that the simple molecules in this study can efficiently form supramolecular polymers in water if they have hydrophobic surfaces larger than about 1 nm^2^. This research illuminates how complex structures can form in the natural world and may have implications for soft-material design and applications. It also provides insights into the origins of genetic materials like RNA and DNA.

Fenniri and coworkers (Fenniri et al., 2001) designed and synthesized a self-assembling molecular system (Figure 1D), with a focus on its unique features and properties. This system is designed to self-assemble in water, and it involves a careful balance between enthalpic losses due to hydrogen bonds and entropic gains from stacking interactions and the hydrophobic effect. The design includes specific molecular components such as a G and C fused base unit with hydrogen bond array and an amino acid moiety to determine the supramolecular chirality.

The existence of inter-modular H-bonds was confirmed through 2D NMR and ESI-MS displayed all peaks related to noncovalent intermediate species. CD spectra illustrated the presence of stacked bases in a helical arrangement, like DNA. NMR, ESI-MS and CD provided comprehensive descriptions of the cooperative and hierarchical self-assembly process driven by hydrogen bonding, stacking interactions, and hydrophobic effects. These assemblies exhibited supramolecular chirality due to helically stacked rosettes.

Characterization techniques, such as DLS and TEM, supported the formation of nanotubular assemblies with specific dimensions. The absence of higher-order twisted or helical aggregates implied the supramolecular chirality is an inherent property of individual nanotubes.

While direct visual evidence of the proposed rosette-stacking mode may be lacking, various observations strongly suggest its prevalence, including MS and NMR data, and TEM results, consistency between calculated and observed nanotube diameters.

Fenniri and coworkers developed a system highlighting the significance of understanding the electrostatic, stacking, and hydrophobic interactions guided by hydrogen bonds in directing the hierarchical assembly of helical nanotubular architectures in an aqueous environment. The authors also raised questions regarding the impact of pH, ionic strength, solvents, amino acids, and peptides on assembly, as well as the potential for further organization into nanoporous materials. Overall, this system offers exciting prospects for designing nanoscale materials with specific dimensions, shapes, and functions.

Chens and Yu (Chen et al., 2017) disclosed a bicyclic system (Figure 1E) with fused A and T. The patent covered the compositions consisting of biomimetic compounds with chemical structures that mimic nucleic acid base pairs. These compounds are made up of two main components: biomimetic adenine-thymine (A^∧^T) and guanine-cytosine (G^∧^C) fused bases or their analogues, and amino acids, polyamino acids, amines, or polyamines. These compounds are used to create nanomaterials that can form various nanostructures like nanotubes, nanorods, nanopieces, and nanosheets. Due to their unique structure, these nanomaterials are suitable for a range of clinical applications, including bio-adhesion and drug delivery, with minimal or no adverse side effects. In the patent, it was claimed that these nanomaterials can be used to introduce therapeutics or diagnostic agents into cells or tissues or tissue matrix and can be used as implant or coating materials.

## 3 Applications

### 3.1 Sensors

**FIGURE 2:**
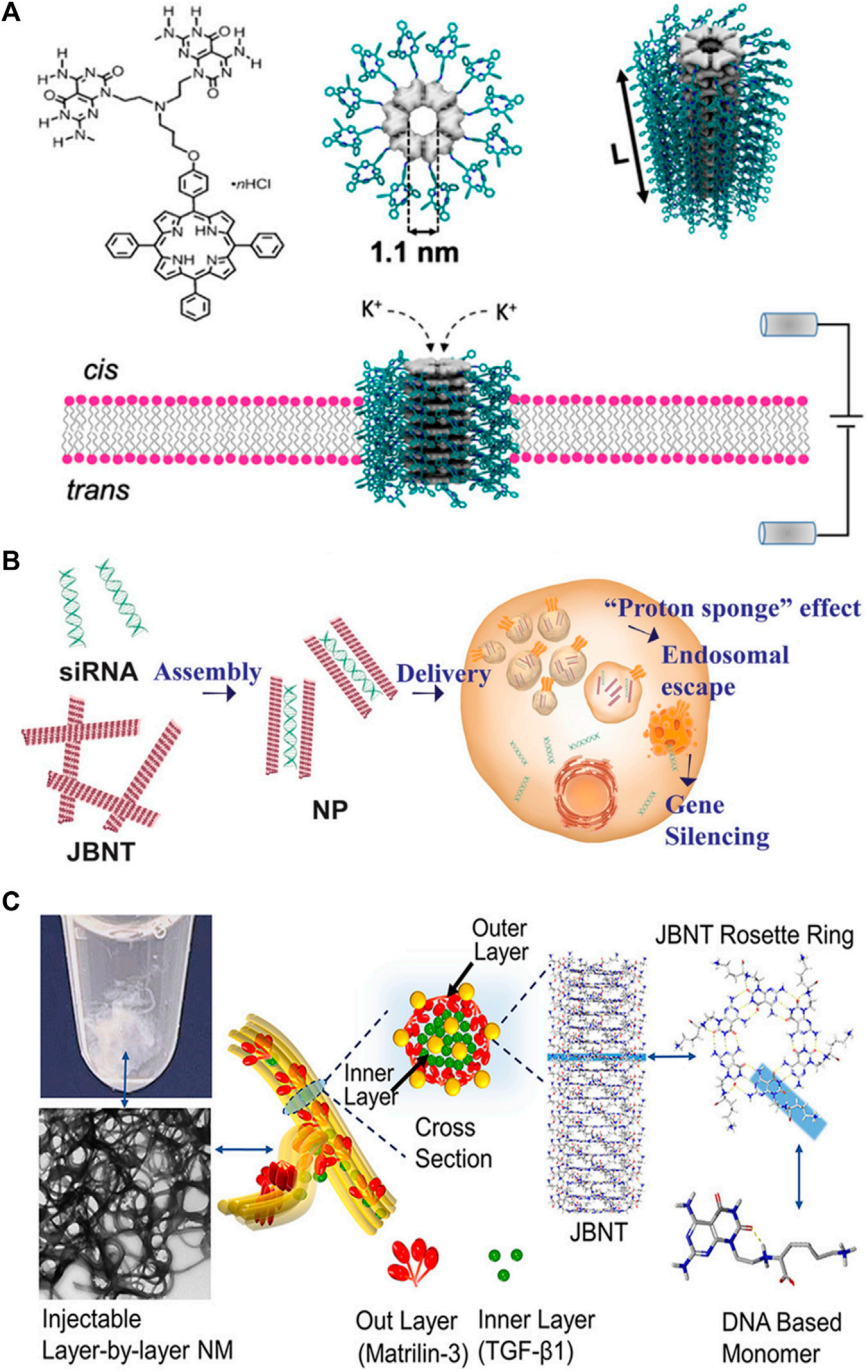
Applications of nanotubes in **(A)** sensors; **(B)** drug delivery; **(C)** tissue regeneration. Reprinted with permission from Tripathi et al. (2020). Rosette Nanotube Porins as Ion Selective Transporters and Single-Molecule Sensors. J. Am. Chem. Soc. 142 (4), 1680–1685. doi: 10.1021/jacs.9b10993. Copyright 2020 American Chemical Society. Adapted from Lee et al. (2021). DNA-inspired nanomaterials for enhanced endosomal escape. Proc. Natl. Acad. Sci. U. S. A. 118 (19), e2104511118. doi: 10.1073/pnas.2104511118. Copyright 2021 National Academy of Sciences. Reprinted from Zhou et al. (2021a). Controlled Self-Assembly of DNA-Mimicking Nanotubes to Form a Layer-by-Layer Scaffold for Homeostatic Tissue Constructs. ACS Appl. Mater Interfaces 13(43), 51321–51332. doi: 10.1021/acsami.1c13345. CC BY-NC-ND 4.0 Deed.

Fenniri and coworkers (Tripathi et al., 2020) recently reported rosette nanotube porins (RNTPs) as ion transporters and single molecule sensors (Figure 2A). To address the solubility challenge, the researchers initially synthesized RNTPs and reconstituted them within lipid vesicles. These vesicles subsequently fused spontaneously with a planar lipid bilayer, resulting in a stepwise increase in ion current across the bilayer.

The study found that most RNTPs inserted into lipid bilayers are short and have similar conductance values to those of biological toxin channels and other artificial channels. These RNTPs exhibited stable ionic currents, making them suitable for single-molecule sensing. However, some RNTPs show unstable and stochastic behavior, which was attributed to mis-assembly or mechanical instability.

The authors also explored the ion selectivity of RNTPs and found no selectivity of K^+^/Cl^−^ at high salt concentrations. This weak selectivity of RNTP toward K^+^ transport was explained through all-atom molecular dynamics (MD) simulations, which revealed the binding of Cl^−^ to positively charged ammonium groups at the junction of G^∧^C bases and porphyrin.

The study further explored the potential of RNTPs as single-molecule sensors using α-cyclodextrins (α-CD). This investigation observed transient blockades in ionic currents and analyzed the interaction between α-CD and RNTPs, revealing two distinct time constants for residence times. Additionally, all-atom MD simulations suggested a mechanism for the variation in open pore values for the three conformations of α-CD at the RNTP.

In conclusion, the study establishes RNTPs as promising *de novo* porins with predefined diameters. It also implies that these new porins could open up new directions, including water purification applications, industrial-scale separations, or flexible reconfigurable nanofluidic circuits.

### 3.2 Drug delivery

Chen and coworkers (Lee et al., 2021) developed a delivery system for RNA interference (RNAi) therapy, addressing the challenges of effective RNA cargo delivery to the cytoplasm and escape from late endosomes (Figure 2B). Current methods, such as lipid nanoparticles (LNPs) and cationic polymers, have limitations, including poor endosomal escape and high cytotoxicity. The researchers developed nanopieces (NPs) based on DNA-inspired Janus base nanotubes (JBNts) to overcome these challenges.

These JBNts could incorporate small interfering RNA (siRNA) through positive–negative charge interactions and base stacking. After loading siRNA, JBNts were processed into NPs, showing stability and effective siRNA encapsulation. Confocal laser-scanning microscopy (CLSM) confirmed the successful intracellular delivery of fluorescently labeled siRNA by NPs. Cellular uptake was energy-dependent, inhibited by low temperature or ATP inhibition, and involved macropinocytosis.

The study demonstrated the NPs’ ability to escape endosomes, attributed to the “proton sponge” effect. The NPs exhibited enhanced endosomal escape compared to LNPs and showed high biocompatibility. The NPs outperformed LNPs in terms of gene silencing efficacy and antiviral potential against a modified adenovirus.

The article highlighted the importance of cytotoxicity in RNAi delivery, emphasizing the superior biocompatibility of JBNt-based NPs compared to LNPs, cationic polymers, and carbon nanotubes. The noncovalent structure and DNA-mimicking chemistry of JBNts contributed to their improved biocompatibility.

In summary, the researchers developed a novel class of delivery vehicles (NPs) based on DNA-inspired Janus bases, demonstrating efficient intracellular delivery, enhanced endosomal escape, and low cytotoxicity. The study presented promising results for potential applications in RNAi therapy, including antiviral treatments.

Webster et al. (2014) claimed an invention that was mainly focused on delivering nucleic acids or polynucleotides into cells using Janus base nanotubes to modulate gene expression or cell function, regulate cell signaling and function, and influence tissue or organ activities. The inventors successfully delivered siRNA, miRNA365, and GAPDH molecular beacons into cells.

Chen and Lee (2022) recently disclosed innovative chemical compositions. Their breakthrough is in the inclusion of formulas that encompass a broader range of elements compared to prior patents. Notably, their patent covers formulas that are chemically linked to therapeutic agents, including small molecules, peptides, nucleic acids, gene editing reagents, or targeting molecules. This expansion in scope yields several noteworthy advantages: 1) Cargoes can be encapsulated through either covalent or non-covalent bonds, offering flexibility in cargo delivery mechanisms; 2) Chemically cleavable linkers can be employed to regulate the controlled release of cargoes, enhancing precision in therapeutic administration; 3) The ability to modify the surface with various targeting molecules facilitates specific and selective uptake pathways. This modification enables the binding of delivery vehicles to specific receptors on cell surfaces, optimizing therapeutic targeting; 4) Coating materials provide protection to the delivery vehicle against both specific and non-specific clearance by cells and organs, enhancing the efficiency of cargo delivery.

The inventors achieved successful delivery of a wide variety of cargos, extending beyond nucleic acids, to encompass small RNAs, proteins, small molecules, and more. Moreover, the applications were also beyond joint and cartilage tissues, for example, they were able to deliver small-molecule chemotherapy drugs for anticancer treatments.

### 3.3 Tissue regeneration

Chen and coworkers (Zhou et al., 2021b) have developed a novel injectable layer-by-layer matrix for cartilage tissue engineering (Figure 2C). The nano-matrix (NM) comprises two distinct layers: an inner layer composed of JBNts enveloped in TGF-β1 and an outer layer consisting of JBNts enveloped in matrilin-3. These layers are assembled through charge interactions, with JBNts carrying a positive charge and matrilin-3 being negatively charged under physiological conditions. The study demonstrated that the NM had excellent structural stability and could prevent TGF-β1 leakage.

The assembly process is both rapid and biomimetic, yielding a solid, porous scaffold structure that closely mimics the extracellular matrix of cartilage. A significant advantage of the NM is its injectability, making it a suitable option for addressing irregularly shaped defects. Furthermore, the exceptional biocompatibility of JBNts is evident, as cell viability remains above 88% even at the highest concentration tested. The NM actively promotes cell anchorage and adhesion, with cells aligning along the scaffold fibers. Moreover, this NM facilitates cell proliferation and chondrogenic differentiation, primarily due to the presence of TGF-β1, matrilin-3, and JBNts. One of its standout characteristics is its superior ability to prevent hypertrophy, a critical aspect of cartilage tissue construct. This achievement is made possible by the stabilization of TGF-β1 within the layer-by-layer structure, ensuring enduring bioactivity.

## 4 Conclusion

Hydrogen bonds have emerged as the cornerstone of these self-assembled nanotubes, facilitating the precise and directional ordering of molecular components. At the same time, the π-π stacking interactions observed in these nanotubes further enhance their stability and structural integrity. These non-covalent forces contribute to the formation of well-defined supramolecular structures with unique physical and chemical properties. Moreover, the ability to self-assemble nanomaterials in water expands their applicability in a wide range of fields, particularly in biomedicine, environmental science, and materials science, while offering advantages in terms of safety, cost, and environmental impact. Further research in this field will undoubtedly uncover new insights and applications, ultimately paving the way for innovative technologies and transformative solutions to some of the most pressing challenges in science and technology.
